# A specific single-stranded DNA induces a distinct conformational change in the nucleoid-associated protein HU

**DOI:** 10.1016/j.bbrep.2016.09.014

**Published:** 2016-10-11

**Authors:** Yuya Nishida, Teppei Ikeya, Tsutomu Mikawa, Jin Inoue, Yutaka Ito, Yasunori Shintani, Ryoji Masui, Seiki Kuramitsu, Seiji Takashima

**Affiliations:** a Graduate School of Frontier Biosciences, Osaka University, 1-3 Yamadaoka, Suita-shi, Osaka 565-0871, Japan; b Department of Medical Biochemistry, Osaka University Graduate School of Medicine, 2-2 Yamadaoka, Suita-shi, Osaka 565-0871, Japan; c Department of Chemistry, Graduate School of Science and Engineering, Tokyo Metropolitan University, 1-1 Minami-Osawa, Hachioji-shi, Tokyo 192-0373, Japan; d CREST, JST, Saitama 332-0012, Japan; e Laboratory for Biomolecular Structure and Dynamics, Cell Dynamics Research Core, RIKEN Quantitative Biology Center, 1-7-22 Suehiro-cho, Tsurumi-ku, Yokohama 230-0045, Japan; f Division of Biology and Geosciences, Graduate School of Science, Osaka City University, 3-3-138 Sugimoto, Sumiyoshi-ku, Osaka-shi 558-8585, Japan; g Department of Biological Sciences, Graduate School of Science, Osaka University, 1-1 Machikaneyama-cho, Toyonaka-shi, Osaka 560-0043, Japan

**Keywords:** NAP, nucleoid-associated protein, TtHU, *Thermus thermophilus* HU, CD, circular dichroism, NMR, nuclear magnetic resonance, ssDNA, single-stranded DNA, dsDNA, double-stranded DNA, HSQC, heteronuclear single quantum coherence, SLBP, stem-loop binding protein., HU, Nucleoid-associated protein, Single-stranded DNA, NMR, Circular dichroism, Thermus

## Abstract

In prokaryotic cells, genomic DNA forms an aggregated structure with various nucleoid-associated proteins (NAPs). The functions of genomic DNA are cooperatively modulated by NAPs, of which HU is considered to be one of the most important. HU binds double-stranded DNA (dsDNA) and serves as a structural modulator in the genome architecture. It plays important roles in diverse DNA functions, including replication, segregation, transcription and repair. Interestingly, it has been reported that HU also binds single-stranded DNA (ssDNA) regardless of sequence. However, structural analysis of HU with ssDNA has been lacking, and the functional relevance of this binding remains elusive.

In this study, we found that ssDNA induced a significant change in the secondary structure of *Thermus thermophilus* HU (TtHU), as observed by analysis of circular dichroism spectra. Notably, this change in secondary structure was sequence specific, because the complementary ssDNA or dsDNA did not induce the change. Structural analysis using nuclear magnetic resonance confirmed that TtHU and this ssDNA formed a unique structure, which was different from the previously reported structure of HU in complex with dsDNA. Our data suggest that TtHU undergoes a distinct structural change when it associates with ssDNA of a specific sequence and subsequently exerts a yet-to-be-defined function.

## Highlights

• We observed the CD spectra and NMR spectra of TtHU bound to various DNA.
• The specific ssDNA affected the secondary structure of TtHU.
• The structure of TtHU bound to ssDNA was distinct from the structure bound to dsDNA.

## 1 Introduction

In prokaryotic cells, genomic DNA forms an aggregated structure with various nucleoid-associated proteins (NAPs) [1]. NAPs have varied structures and hence diverse functions [2], [3]. The functions of genomic DNA, such as replication, segregation, translation and repair, are related to its distinct structure, which is cooperatively modulated by NAPs [4], [5], [6], [7].

HU (H protein from *Escherichia coli* U93) is the most conserved and the most abundantly expressed NAP [8], [9], [10]. In some bacteria, mutation in the HU gene and gene disruption of HU affect cell growth or is lethal [11], [12]. These results have suggested that HU has a central role among NAPs. HU is a small protein consisting of approximately 90 amino acid residues and mainly exists as a dimer in solution [13]. It has been reported that the interaction between double-stranded DNA (dsDNA) and HU is non-specific [14], [15]. The binding of HU leads to a bent and a negative supercoiling in the dsDNA structure [16], [17]. Some structures of HU alone and in complex with dsDNA have been determined [18], [19], [20] and have shown that HU has two beta-arms and grips the dsDNA by engagement of the arms in the minor groove. HU also serves as an structural modulator of dsDNA architecture and plays important roles in DNA replication, segregation, repair and transcription [11], [12], [21], [22], [23], [24], [25].

Interestingly, it has been reported that HU can also bind single-stranded DNA (ssDNA), and this interaction is also non-specific [15], [26], [27]. ssDNA intermediates are created by DNA unwinding and serve as template for DNA replication or repair processes. However, little is known of the interaction between HU and ssDNA. Structural information has not been obtained, and the functional relevance of this binding remains elusive.

In this study, we used HU from *Thermus thermophilus* HB8 (TtHU). To characterize the structure of TtHU bound to ssDNA, we performed circular dichroism (CD) spectral analysis and nuclear magnetic resonance (NMR) spectral analysis. Our data suggest that ssDNA of a specific sequence induces a significant structural change in the secondary structure of TtHU, which is different from the change shown previously in HU bound to dsDNA.

## 2 Materials and methods

### 2.1 Materials

The sequences of the chemically synthesized ssDNA (BEX Co., Ltd. or FASMAC Co., Ltd.) are described in Fig. 2I. dsDNA oligo AB and oligo CD were prepared by incubation of ssDNA oligos at 95 °C for 10 min, and the temperature was then decreased at a rate of 1 °C per min to anneal.

### 2.2 Purification of TtHU

*E. coli* BL21(DE3) was transformed with TtHU/pET-11a and grown at 37 °C in LB medium containing 50 μg/mL ampicillin. When the culture reached log phase, IPTG was added to 50 μg/mL. Cells were grown for 12 h after induction and harvested by centrifugation. Cells were suspended in 20 mM Tris–HCl (pH 7.8), 500 mM NaCl and 5 mM EDTA. The cells were disrupted by sonication and then heated at 70 °C for 20 min. After centrifugation at 22,500*g* for 1 h, the clear supernatant was loaded onto a Toyopearl SP-650 M column (Tosoh) equilibrated with 20 mM Tris–HCl (pH 7.8), 500 mM NaCl and 5 mM EDTA. The column was washed with the buffer and eluted with a gradient of 500–1500 mM NaCl in the buffer. The fractions containing TtHU were detected by SDS-PAGE and concentrated using a Vivaspin 20–10 K (MWCO 10,000 Da, GE healthcare) concentrator. The solution was then applied to a HiLoad 16/60 Superdex 75 column (Tosoh) equilibrated with 20 mM Tris–HCl (pH 7.8) and 2 M NaCl and eluted with the same buffer. Purified proteins were stored in 20 mM Tris–HCl (pH 7.8) and 150 mM NaCl at 4 °C.

### 2.3 Electrophoretic-mobility shift assay

Chemically synthesized oligo DNAs were incubated with various concentrations of TtHU in 20 mM Tris–HCl (pH 7.5) and 100 mM KCl at 37 °C for 1 h. The mixtures were loaded onto a polyacrylamide gel and electrophoresed in TBE buffer (pH 8.2, 89 mM Tris-borate and 2 mM EDTA). The bands were visualized with GelRed (Wako) and UV irradiation.

### 2.4 Circular dichroism structural analysis

CD spectra were recorded with a JASCO-720W spectropolarimeter, with a 0.1 cm cuvette at 20 °C for 200–300 nm. In the titration analysis, 40 µM of DNA solution was added to 300 μL of 10 µM TtHU solution (pH 7.2, 20 mM phosphate and 100 mM KCl). The effect of increasing volume due to titration was calculated after the experiment.

### 2.5 Purification of TtHU for NMR

*E. coli* Rosetta2(DE3) was transformed with TtHU/pET-11a and grown in M9 medium (0.6% Na_2_HPO_4_, 0.3% NaH_2_PO_4_, 0.05% NaCl, 0.1% NH_4_Cl, 0.2% glucose, 2 mM MgSO_4_·7H_2_O, 0.1 mM CaCl_2_·2H_2_O, 33 µM FeCl_3_·6H_2_O, and 50 μg/mL ampicillin, pH 7.2) containing [^15^N]NH_4_Cl and/or [^13^C]glucose as the sole nitrogen and/or carbon source for labelled ^15^N-TtHU and/or ^13^C/^15^N-TtHU, respectively. The purification procedure for labelled TtHU was the same as that for the unlabelled TtHU.

### 2.6 NMR data collection

The NMR sample contained 1 mM TtHU, 20 mM phosphate (pH 7.2) and 100 mM KCl. The NMR spectra were measured at 303 K by using a Bruker Avance III 600 MHz spectrometer equipped with a cryogenic TCI probe head. The sequence-specific backbone ^1^H^N^, ^13^C_α_, ^13^C′, and ^15^N and side chain ^13^C_β_ resonance assignments of ^13^C/^15^N-labelled TtHU were obtained from CBCA(CO)NNH, CBCANNH, HNCO, and HN(CA)CO experiments [28], [29]. Data were processed using CcpNmr Analysis [30].

### 2.7 NMR structural analysis of TtHU's DNA binding

The two-dimensional heteronuclear single quantum coherence (HSQC) spectra were acquired with^15^N-labelled TtHU in the presence of various concentrations of oligo A. In this study, a concentrated oligo A solution was added to a 0.1 mM ^15^N-labelled TtHU solution to prevent changes in concentration.

### 2.8 Construction of a model structure of TtHU

The model structure of TtHU was constructed on the basis of a previous structure of HU from *Staphylococcus aureus* (PDB: 4QJU), using ROBETTA (http://robetta.bakerlab.org/).

## 3 Results

### 3.1 The effect of ssDNA on the secondary structure of TtHU

Although it has previously been reported that HU can bind to ssDNA as well as dsDNA in a sequence-independent manner [14], [15], little is known about the interaction between HU and ssDNA. In particular, structural analysis has been lacking.

**Fig. 1:**
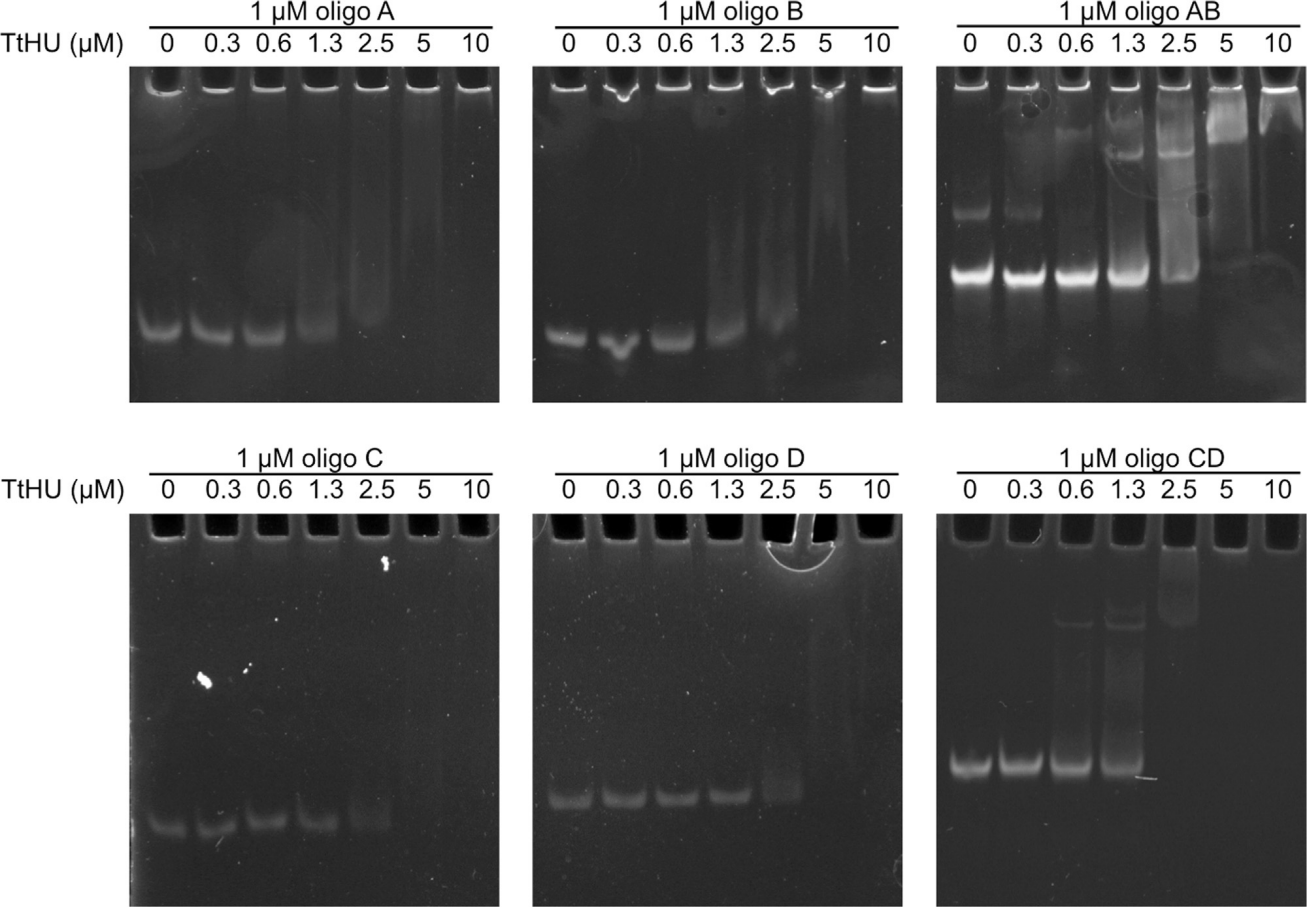
Analysis of the binding between TtHU and ssDNA or dsDNA. TtHU was incubated with 1 µM of each oligo DNA. Each mixture was electrophoresed and visualized with GelRed.

In this study, we chose 2 sets of complementary ssDNAs that we usually use as controls for gel shift assays in our laboratories: partial oligonucleotides from beta-lactamase (oligos A and B, which are complementary to each other) and from beta-galactosidase (oligos C and D, also complementary). TtHU bound to all those 4 ssDNAs as well as to their complementary dsDNAs (Fig. 1), in agreement with earlier reports [14], [15], [26].

**Fig. 2:**
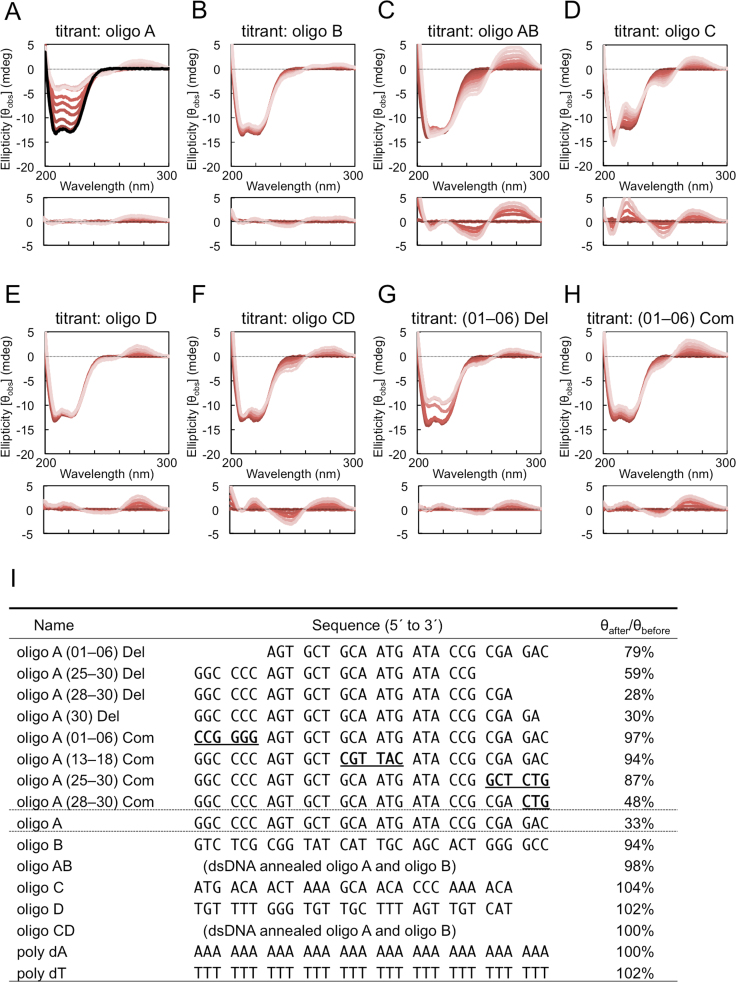
A specific ssDNA induced a change in the CD spectra of TtHU. CD spectra were observed in titration analyses with various DNAs. The titrant solutions were 40 µM of (A) oligo A, (B) oligo B, (C) oligo AB, (D) oligo C, (E) oligo D, (F) oligo CD, (G) oligo A (01-06) Del, or (H) oligo A (01-06) Com, as indicated above the parts. The sequences of the oligonucleotides are described in (I). The titrand solution was 10 µM HU (top) or buffer (bottom). The spectrum before titration is shown as a dark red line, and the colour is reduced with increasing concentration of DNA from 0 µM to 10 µM. The black line in (A) shows the results before titration. (I) The ratio of the CD value after titration (θ_after_) to the CD value before (θ_before_). The ratios were calculated by subtracting the value at 222 nm of HU solution as titrand from the CD value of the buffer solution.

Next, to evaluate the effect of ssDNA binding on the structure of TtHU, we performed secondary structural analysis of TtHU by using CD spectra. As shown in Fig. 2A (top, black line), the CD spectrum of TtHU had a maximal negative signal at approximately 210–220 nm. This result was consistent with the structures of HU determined by X-ray crystallography, which showed that HU consists of an alpha-helix core [19], [20]. We sequentially added various DNA solutions to the TtHU solution and measured their CD spectra. The CD spectra of DNA without HU showed typical positive peaks at 220 nm and 280 nm and negative peaks at 210 nm and 250 nm (bottom spectra in all parts of Fig. 2). Interestingly, when oligo A was added to the TtHU solution, the intensity of the negative peaks at approximately 210–220 nm decreased to one-third of the intensity before DNA addition, as shown in Fig. 2A. In contrast, when the complementary oligo B or double-stranded oligo AB was added, no significant spectral change was observed (Figs. 2B and 2C). Further, oligos C and D and the double-stranded oligo CD did not cause a similar spectral change (Figs. 2D, 2E and 2F). We also performed the same experiments using 30 nt polydeoxyadenosine (poly dA) and 30 nt polydeoxythymidine (poly dT), which were non-self-structured oligonucleotides, but no significant change was observed (Fig. 2I).

Next, we tested several oligo DNAs with some variations in the sequence of oligo A by CD analysis. Whereas deletion of the 5´- or 3´-terminal stretches resulted in a partial loss of the effect of oligo A (Figs. 2G and 2I), complementary sequence-exchange almost completely cancelled the effect of oligo A (Figs. 2H and 2I), thus suggesting that oligo A has a unique sequence-specific effect on the CD spectrum with TtHU. These results led us to hypothesize that ssDNA of a specific sequence unfolds the secondary structure of TtHU, especially in the alpha-helical core, or induces changes in the conformation of TtHU. It is possible that the CD spectral change observed in this study was caused by the ssDNA. However, previously reported CD spectral changes of DNA due to both conformational changes of DNA and binding of proteins are smaller than the changes observed in this study [31], [32]. The decrease in the CD spectrum was observed specifically for oligo A but not for oligo B or oligo AB, though TtHU bound similarly to all of them. Thus, it is unlikely that the decrease in the CD spectrum was caused by the aggregation of TtHU.

### 3.2 NMR structural analysis

**Fig. 3:**
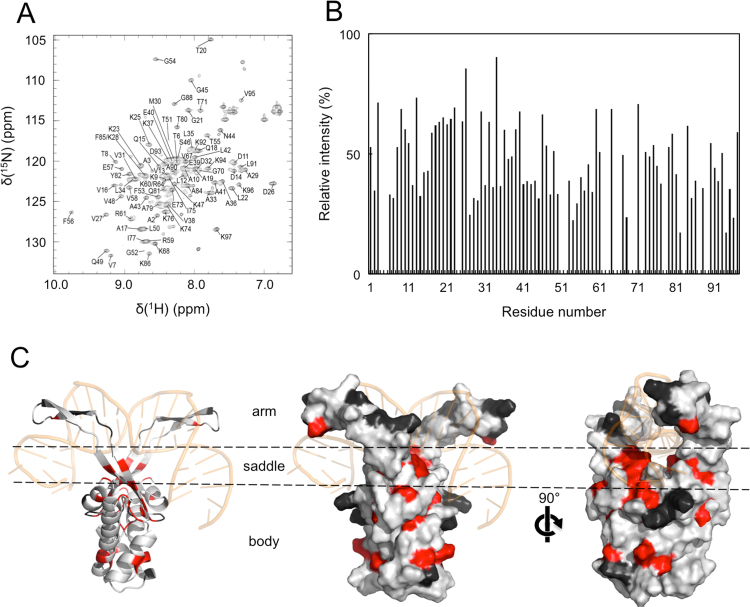
NMR analysis of TtHU binding to oligo A. (A) The ^1^H–^15^N HSQC spectrum of TtHU at a protein concentration of 1 mM TtHU with sequence assignment. (B) Relative intensities of NMR spectra of TtHU with 10 µM oligo A, normalized to the intensity of free TtHU. (C) The residues affected by oligo A binding. The residues with relative intensities of less than 35% are mapped on the model structure of TtHU in red. Unassigned residues are shown in black. The structures are shown as a cartoon representation (left) or surface representation (right).

To further investigate the effect of oligo A on the structure of TtHU, we performed NMR spectral analysis. By analysing the three-dimensional NMR spectra using TtHU alone, we successfully achieved sequential assignment for 91% of non-proline residues in TtHU (Fig. 3A). As shown in Fig. 3C, the unassigned residues were clustered on the beta-arms of TtHU, suggesting that those residues were less stable. This result was consistent with those of previous structural analyses showing flexibility of the beta-arms in HU [19], [33].

To analyse the effect of oligo A on the structure of TtHU, we acquired the HSQC spectra of TtHU with and without oligo A. The intensity of the HSQC peaks with oligo A was decreased compared with the intensity without oligo A, thus suggesting that oligo A induced a significant change in the structure of TtHU. To identify the amino acid residues important for interaction with ssDNA, we sequentially added oligo A at low concentrations, from 0 µM to 20 µM, and acquired the HSQC spectra. Comparison of the HSQC spectra for different concentrations of oligo A revealed that almost all HSQC peaks for each amino acid residue decreased, but the extent varied. This result indicated that some residues were affected strongly by oligo A binding, whereas others were less affected.

To further elucidate the effect of oligo A on TtHU, the intensity of HSQC peaks after the titration of 10 µM DNA was normalized to the intensity before titration (Fig. 3B). Then, the amino acid residues with the relative intensity of less than 35% were plotted on the structure of TtHU constructed by homology modelling (Fig. 3C). These results show that the residues around the saddle region were strongly affected by oligo A binding. Interestingly, though the beta-arms of TtHU are reportedly important for dsDNA binding, the residues in red were not often observed around the arms in this study. Instead, ssDNA induced a strong effect on residues of the body, far from the beta-arms. These results suggest that TtHU bound to ssDNA of a specific sequence forms a distinct conformation from that of the complex with dsDNA.

## 4 Discussion

Few studies have focused on the interaction between HU and ssDNA, and no structural information on ssDNA-bound HU has previously been reported [15], [26], [27]. Here, we report that, in addition to the well-known structure with dsDNA, TtHU undergoes a distinct structural change when it associates with ssDNA of a specific sequence.

**Fig. 4:**
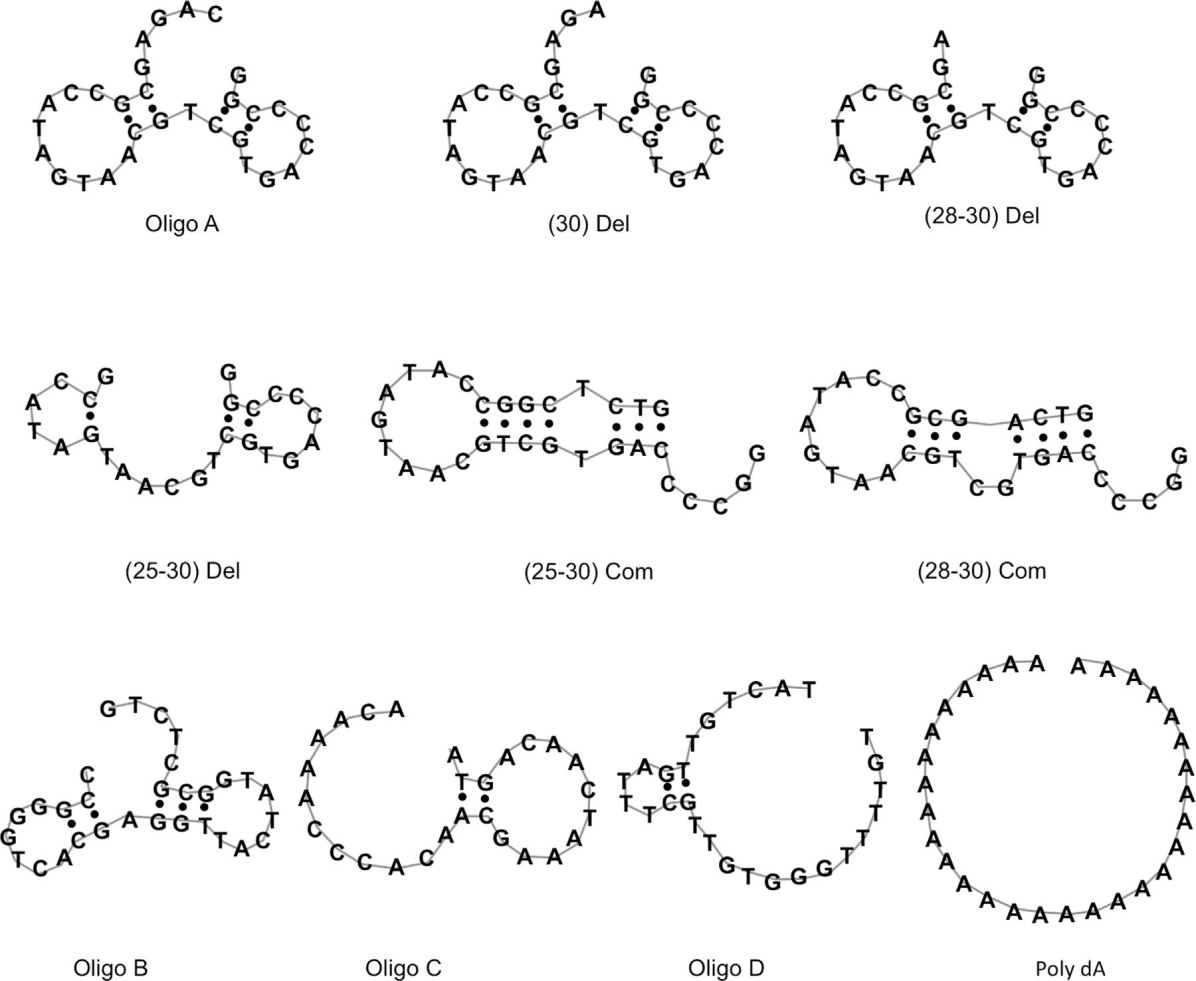
The predicted secondary structures of the oligo DNAs. Structure prediction was performed by using the MaxExpect web server.

We supposed that the effect of the ssDNA (oligo A) depends on its structure, as nucleotide deletion did not cause the same effect as nucleotide exchange (for example, oligo A (25–30) Del vs oligo A (25–30) Com). Thus, we predicted the secondary structure of the oligo DNAs by using a program MaxExpect web server [34], [35]. As shown in Fig. 4, Oligo A is predicted to have two small stem-loops which are bridged by short and nicked double-stranded DNA (dumbbell shape). Similar structures are also found in the predicted structures of oligo A (28–30) Del and oligo A (30) Del, both of which showed compatible effect on HU as oligo A did. The deletion or exchange in oligo A that lost the effect on HU are predicted to be destructive on the stem-loops in oligo A. For example, oligo A (25–30) Del is predicted to have two stem-loops and retains the effect on HU (59%), although oligo A (25–30) Com, which is predicted to have only a stem-loop and long double-stranded region, has little effect on HU. In the case of the DNAs which partially maintain the effect, the secondary structures might be mixed probably due to instability of the structure. Meanwhile, the DNAs, which did not have any effect on HU (Oligo C, D and poly dA), are predicted to have long single-stranded region which is distinct from the structure predicted for oligo A. Oligo B is predicted to have a similar structure with oligo A, although the loop size is slightly different. Despite of its similarity to oligo A in the predicted structure, the nucleotide base composition of oligo B is different from that of oligo A as it is complementary to oligo A. From these results, we suppose that the combination of structure and sequence differences might contribute to the specificity of the structural change of HU.

HU plays various important roles in DNA replication, segregation, repair and transcription [11], [12], [21], [22], [23], [24], [25], but the molecular details are still unclear. HU binds dsDNA in a sequence-independent manner and induces a bent and a negative supercoiling in the dsDNA structure, leading to the condensed structure of the nucleoid [8], [15], [16], [17]. It has been suggested that the negative supercoiling induced by HU also resolves the distortion induced by replication and transcription, and thus HU indirectly promotes replication and transcription [4], [16], [17]. From our study, we presume that HU forms a distinct structure with untwisted ssDNA which forms some unique stem-loop structure with similarity to oligo A, as suggested by our results. In contrast to the indirect supercoiling-mediated effect of HU bound to dsDNA, such structure might directly help replication or transcription properly undergo. Stem-loop binding protein (SLBP) is one of the well-known proteins that recognize the stem-loop with a sequence and structure specificity [36], [37]. As SLBP recognizes 26-nt stem-loop structure, our assumption that HU recognizes the unique stem-loop structure seems reasonable.

In conclusion, our data suggest that TtHU undergoes a distinct structural change when it associates with ssDNA of a specific sequence and a specific structure, thereby producing functional diversity. Thus far, structural research on HU has been based on the co-crystal structure of HU bound to dsDNA, and further structural analysis should provide novel insight into the diverse functions of HU with nucleic acids.
